# Effect of Oxygen on Glucose Metabolism: Utilization of Lactate in *Staphylococcus Aureus* as Revealed by *In Vivo* NMR Studies

**DOI:** 10.1371/journal.pone.0058277

**Published:** 2013-03-05

**Authors:** Maria Teresa Ferreira, Ana S. Manso, Paula Gaspar, Mariana G. Pinho, Ana Rute Neves

**Affiliations:** 1 Laboratory of Lactic Acid Bacteria & in vivo NMR, Instituto de Tecnologia Química e Biológica, Universidade Nova de Lisboa, Oeiras, Portugal; 2 Laboratory of Bacterial Cell Biology, Instituto de Tecnologia Química e Biológica, Universidade Nova de Lisboa, Oeiras, Portugal; University of Liverpool, United Kingdom

## Abstract

The ability to successfully adapt to changing host conditions is crucial for full virulence of bacterial pathogens. *Staphylococcus aureus* has to cope with fluctuating oxygen concentrations during the course of infection. Hence, we studied the effect of oxygen on glucose metabolism in non-growing *S. aureus* COL-S cells by *in vivo* ^13^C-NMR. Glucose catabolism was probed at different oxygen concentrations in suspensions of cells grown aerobically (direct effects on metabolism) or anaerobically (transcriptional adjustment to oxygen deprivation). In aerobically-grown cells, the rate of glucose consumption diminished progressively with decreasing oxygen concentrations. Additionally, oxygen deprivation resulted in biphasic glucose consumption, with the second phase presenting a higher rate. The fructose-1,6-bisphosphate pool peaked while glucose was still abundant, but the transient maximum varied with the oxygen concentration. As oxygen became limiting mannitol/mannitol-1-phosphate were detected as products of glucose catabolism. Under anoxic conditions, accumulation of mannitol-1-phosphate ceased with the switch to higher glucose consumption rates, which implies the activation of a more efficient means by which NAD^+^ can be regenerated. The distribution of end-products deriving from glucose catabolism was dramatically affected by oxygen: acetate increased and lactate decreased with the oxygen concentration; ethanol was formed only anaerobically. Moreover, oxygen promoted the energetically favourable conversion of lactate into acetate, which was particularly noticeable under fully oxygenated conditions. Interestingly, under aerobiosis growing *S. aureus* cells also converted lactate to acetate, used simultaneously glucose and lactate as substrates for growth, and grew considerably well on lactate-medium. We propose that the efficient lactate catabolism may endow *S. aureus* with a metabolic advantage in its ecological niche.

## Introduction

*Staphylococcus aureus* is a Gram-positive bacterium frequently found on the skin and in the nasal cavity of humans. Even though about 20% of the human population are long-term carriers and 60% are intermittent carriers of *S. aureus,* this extremely versatile pathogen is better known as a major cause of hospital and community acquired diseases, which range from minor skin infections to life-threatening endocarditis, meningitis or sepsis [1]; [2]. With widespread bacterial antibiotic resistance, of which *S. aureus* is a paradigm due to its remarkable adaptive capacity, it is urgent to find new ways to combat staphylococcal infections. One such opportunity is offered by the recognition of overlapping networks between pathogenesis and basic physiology. Fully corroborating this view, a number of studies have implicated central metabolic pathways in regulating or affecting staphylococcal virulence [3]–[7]. Thus, the fitness of *S. aureus* in the host, namely its ability to successfully adapt to different niches, is essential for virulence. Consequently, elucidation of the underlying metabolic and regulatory networks involved is mandatory for an in-depth understanding of staphylococcal pathogenesis.

A main environmental factor varying during the course of infection is the oxygen availability. As *S. aureus* migrates from residing on the skin or nasal cavity to being embedded in internal host niches, the availability of free oxygen noticeably diminishes: from the atmospheric partial pressure of 159 mm Hg (aerobic) to an estimated 3–5 mm Hg (hypoxic, dissolved oxygen concentration below 10 µM) at the host cell level.

*S. aureus* is a facultative anaerobe that possesses the Embden-Meyerhof-Parnas (EMP), the pentose phosphate (PPP) and the tricarboxylic acid cycle (TCA) pathways for the catabolism of carbohydrates. Reports from the 1960′s proposed that the activity of the TCA cycle is largely dependent on environmental conditions and nutrient availability [8]; [9], observations that have been fully corroborated by a number of recent studies [5]; [10]–[13]. Oxygen deprivation was pinpointed as one of the key conditions antagonistic of TCA cycle activity [9]; [14]. In line, when subjected to a switch from aerobic to anaerobic, *S. aureus* decreases the transcript levels of the pyruvate dehydrogenase complex and TCA cycle genes and up-regulates genes involved in glycolysis [10]. The major implication of this physiological response to oxygen is a dramatic shift in central metabolism, from the energy favorable respiratory state to a fermentative condition, in which lactate is the major end-product. Fairly good descriptions of extracellular metabolite dynamics during anaerobic and aerobic glucose metabolism are available [9]; [10]; [12]–[17], but little is known about the pools of intracellular metabolites in *S. aureus*. Considering the central role of metabolic intermediates in regulatory processes, in which they can intervene directly by modulating enzyme activity, or in gene regulation as cofactors of regulatory proteins, it is imperative to obtain reliable data on intracellular metabolite concentrations.

Fostered by this gap of knowledge, we envisaged using *in vivo* NMR to obtain time series data on intracellular metabolites in *S. aureus*. This technique has been successfully applied to examine the effect, at the metabolic level, of environmental and genetic perturbations in a number of different microorganisms, including *S. aureus* (reviewed in [18]–[20]). To investigate the effect of oxygen on glucose metabolism of non-growing cells of *S. aureus* COL-S in a non-invasive manner by NMR, we conceived an experimental design that allowed discriminating between direct effects of oxygen on metabolite pools and transcriptional effects: glucose catabolism was probed at different oxygen concentrations (anoxic, semi-aerobic or fully oxygenated) in suspensions of aerobically-grown cells (direct effects) or cells grown anaerobically (transcriptional adjustment to oxygen deprivation). For each condition assayed the concentrations of glucose, phosphorylated intermediates, and the intracellular and extracellular end-product pools were obtained by ^13^C-NMR. The finding that oxygen dramatically potentiated lactate catabolism in cell suspensions led us to survey the potential of *S. aureus* to use and grow on this organic acid. The implications of our findings are discussed in terms of fitness in its ecological niche, the human host.

## Results

### A Chemically Defined Medium Improved for *in vivo* NMR Studies Sustains Aerobic and Anaerobic *S. aureus* Growth

**Figure 1:**
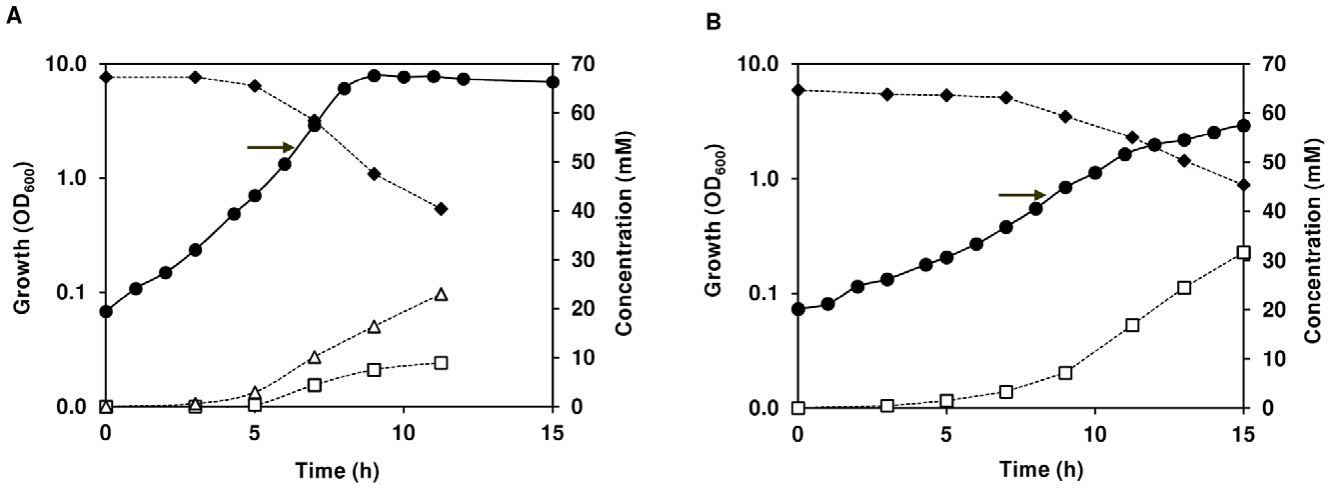
Growth profile of *S. aureus* COL-S in glucose-CDM. Growth, glucose consumption, and end-product formation by *S. aureus* COL-S under aerobic (A) and anaerobic (B) conditions. Symbols: (black diamond), glucose; (open square), lactate; (open triangle), acetate; (black circle), biomass. At the time indicated by the arrow a culture sample was withdrawn for *in vivo* NMR analysis. Data from a representative experiment where the error in each point ≤10%.

The improved chemically defined medium (CDM) for *in vivo* NMR (for details see Materials & Methods) is a phosphate buffered nutritionally rich medium containing 20 amino acids, 10 vitamins, 3 nucleosides, acetate, citrate, a few minerals and glucose as main carbon source. To ensure high quality spectral data the CDM is devoid of paramagnetic ions (e.g. Fe^2+^, Fe^3+^, Mn^2+^), which are common ingredients in CDM for *Bacilli*. The profiles of growth for strain COL-S, a methicilin sensitive derivative of the MRSA *S. aureus* COL strain, were obtained under aerobic and anaerobic conditions (Fig. 1).

Cultivation of *S. aureus* was performed in CDM supplemented with glucose (65 mM), at 37°C, and without pH control (initial pH 7.0), in shake flasks agitated at 200 rpm (aerobic conditions) or rubber-stoppered standing bottles (anaerobic conditions). In the presence of oxygen, the maximal growth rate was about 1.1 h^−1^ (doubling time of 58 min), and the stationary phase was reached at an OD_600_ of 7.5 after 9 hours of growth (Fig. 1A). To ensure that the cultivations in shake flasks (1/5 volume to flask ratio) yielded aerobic growth, cells were also grown in 1/5 volume to flask ratio in baffled flasks or in 1/8 volume to flask ratio in normal flasks. No differences in growth profiles, maximal growth rate (µ_max_) or biomass were observed between the three conditions tested, and thereby the 1/5 volume to flask ratio at 200 rpm warrants aerobic cultivation. Under anaerobic conditions, *S. aureus* showed substantially slower growth (doubling time 126 min), as well as significantly reduced biomass when it reached stationary phase (OD_600_ of 2.4, Fig. 1B).

Under glucose excess, aerobically growing *S. aureus* secreted acetate and lactate to the medium in a proportion of 2∶1, respectively (Table 1). Of the consumed glucose, about 60% was recovered as secreted products in culture supernatants. At the time of growth arrest, 26% of the initial glucose had been consumed and the maximal consumption rate was 12.4 mmol g^−1^ h^−1^. Under anaerobic conditions, a typical homolactic fermentation profile was observed, the carbon balance was above 80%, the glucose consumed amounted to 30% of the total, and the maximal substrate consumption rate increased 1.6-fold relatively to aerobic growth.

**Table 1:** Product yields, carbon and redox balances, growth and energetic parameters, total substrate and pH values determined at the time-point of maximal biomass achieved by *S. aureus.*

	Anaerobic	Aerobic			
Substrate(initial conc. mM)	Glc(63.9±1.1)	Glc(67.9±0.9)	Glc^c^(6.3±0.2)	Glc+Lct^d^(6.3±0.2)	Lct(58.7±0.7)
				(23.0±2.4)^d^	
**Product yields**					
Lactate	1.64±0.00	0.39±0.02	0.07±0.00	NA	NA
Acetate	BDL	0.84±0.01	1.22±0.06	1.39±0.02	0.78±0.01
**q_s_^max^ (mmol g** ^−**1**^ ** h** ^−**1**^ **)**	19.9±1.7	12.4±0.2	8.6±0.1	15.7±0.1	34.2±3.0
				28.1±3.0^d^	
**µ_max_ (h** ^−**1**^ **)**	0.47±0.01	1.05±0.08	1.00±0.07	1.05±0.02	0.72±0.02
**Doubling time (min)**	126±2	58±4	60±1	58±3	84±2
**Max(OD_600_)**	2.4±0.1	7.5±0.4	6.1±0.1	5.2±0.2	6.7±0.2
**Carbon balance** a	82±0	61±2	64±2	70±1	78±1
**Redox balance** b	82±0	NA	NA	NA	NA
**Biomass yield (g mol** ^−**1**^ ** of substrate)**	19.2±0.0	60.0±2.2	98.4±0.6	31.3±2.6	27.2±2.0
**ATP yield (mol mol** ^−**1**^ ** of substrate)**	1.6±0.0	NA	NA	NA	NA
**YATP (g of biomass mol** ^−**1**^ ** of ATP)**	11.7±0.0	NA	NA	NA	NA
**Consumed substrate (%)**	30±1	26±4	100	100	60±1
				76±2^d^	
**pH (growth arrest)**	6.6±0.2	7.3±0.2	7.2±0.00	6.8±0.02	7.9±0.1

COL-S cultured on glucose and/or lactate. Substrate consumption rate and the specific growth rate are also shown. Values of two or three independent experiments were averaged and errors are reported as ±SD.

a Carbon balance is the percentage of carbon in metabolized Glc or Lac that is recovered in the fermentation products (lactate, formate, ethanol and acetate);

b Redox balance is the ratio between [lactate]+2×[ethanol] and 2×[Glc] multiplied by 100.

c Carbon balance and biomass yield were calculated for time-point of glucose depletion (t7).

d Data referring solely to lactate consumption; q_s_^max^ was estimated from a first-order derivative of a polynomial fit of the observed substrate consumption time series. Dry weight (DW) was used as a measure of cell mass. BDL, below detection limit. NA, not applicable; Glc, glucose; Lct, lactate.

In summary, our glucose-CDM sustained high biomass production of *S. aureus* under aerobic growth. Unsurprisingly, growth parameters were negatively affected by anaerobic conditions, but the biomass yield was still in the range required to prepare dense cell suspensions for *in vivo* NMR studies.

### Oxygen Dramatically Changes Glucose Metabolism of Aerobically Grown *S. aureus* Cells

In the human body *S. aureus* is exposed to fluctuating oxygen environments. Quick responses to sudden changes in environmental conditions often occur at the metabolic level. Hence, we deemed it important to examine how the central carbon pathways respond to oxygen availability.

The effect of oxygen on glucose metabolism was studied by *in vivo* ^13^C-NMR using dense suspensions (OD_600_ of 200) of *S. aureus* COL-S cells cultivated aerobically and harvested in exponential growth phase (OD_600_ of 2.2, arrow in Fig. 1A). In our experimental set-up oxygen availability was modulated by bubbling through the suspension different gases: pure oxygen (aerobic metabolism), air (semi-aerobic or oxygen limited metabolism), and argon (anaerobic metabolism).

#### Aerobic glucose metabolism

**Figure 2:**
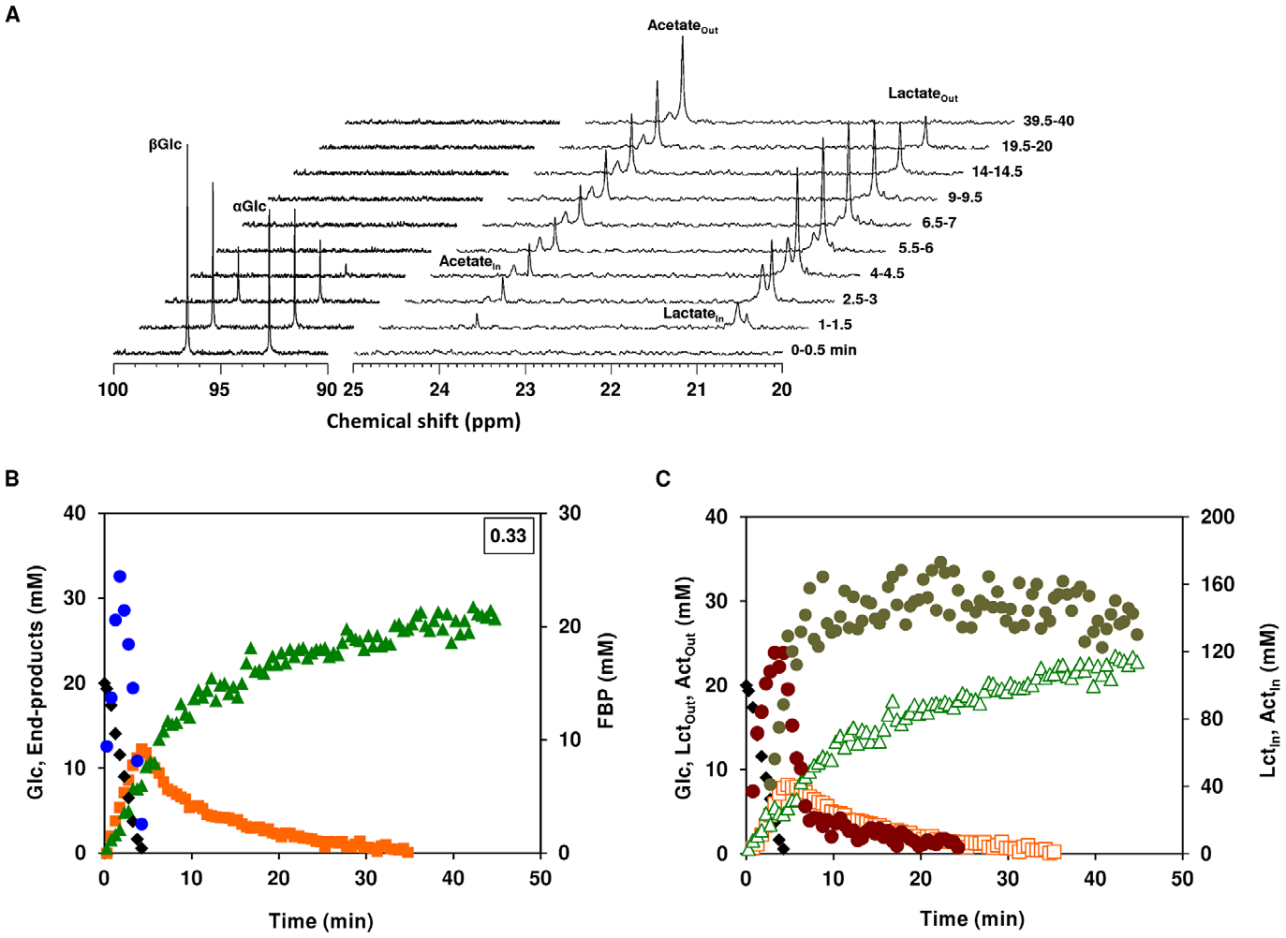
Aerobic glucose metabolism in aerobically-grown *S. aureus* as monitored by *in vivo* ^13^C-NMR. Sequence of ^13^C-NMR spectra acquired during the metabolism of 20 mM [1-^13^C]glucose by a cell suspension of COL-S under aerobic conditions at 37°C. Cells were suspended in 100 mM KP_i_ pH 7.0, at a concentration of 30 mg of DW ml^−1^. Each spectrum represents 30 s of acquisition. Glucose was added at time zero, each spectrum was acquired during the indicated interval, and processed with a 2 Hz line broadening (A). Kinetics of [1-^13^C]glucose (20 mM) consumption, end-products formation and phosphorylated intermediates in *S. aureus* COL-S. Maximal glucose consumption rates (µmol min^−1^ mg^−1^ of protein) are boxed in the upper-right corners (B). Dynamics of intra- and extracellular pools of lactate and acetate; Glc is also shown for the sake of clarity (C). Symbols: (black diamond), glucose; (orange square), total lactate; (green triangle), total acetate; (blue circle), FBP; (open orange square) extracellular lactate; (brown circle) intracellular lactate; (open green triangle) extracellular acetate; (olive circle) intracellular acetate. Total lactate and acetate are the sum of extra- and intracellular concentrations without correcting the latter for the internal volume.

A typical sequence of ^13^C-spectra (regions 90–100 and 20–25 ppm) acquired during the aerobic metabolism of [1-^13^C] glucose (20 mM) by *S. aureus* COL-S is depicted in Fig. 2A. Under these conditions (aerobic growth, aerobic suspensions), resonances due to extracellular glucose (α- and β-anomers at δ 96.7 and δ 92.9 ppm, respectively), lactate (δ, 20.6 ppm) and acetate (δ, 23.8 ppm) were detected. The two resonances at 20.7 ppm and 24.0 ppm downfield of lactate and acetate were assigned to intracelullar lactate and acetate, respectively (Fig. 2A). Additionally, resonances due to the glycolytic metabolite fructose-1,6-bisphosphate (FBP) labelled on C-1 (δ, 66.4 ppm; [1-^13^C]FBP) or C-6 (δ, 65.1 ppm; [6-^13^C]FBP) were present, but only while glucose was available (not shown). FBP is labelled on C-1 or C-6 due to scrambling of the label at the level of triose-phosphate isomerase and backflux through aldolase.

Glucose was consumed at maximal rate of 0.33±0.03 µmol min^−1 ^mg^−1^ of protein, yielding lactate and acetate (Fig. 2B). The glycolytic intermediate FBP accumulated immediately after glucose addition, reached a transient maximal concentration of 24±2 mM when glucose was about mid-way, and decreased to undetectable levels before glucose depletion. At this point, both the intracellular and extracellular pools of lactate reached their maximal concentrations (Fig. 2C), and subsequently declined to exhaustion. However, while the extracellular pool disappeared non-linearly in a slow fashion (maximal consumption rate below 0.04 µmol min^−1^ mg^−1^ of protein), the intracellular lactate pool suffered a sharp linear decrease (consumption rate 2.5 µmol min^−1 ^mg^−1^ of protein) from about 120 to 20 mM, followed by slow depletion. These profiles might indicate a bottleneck at the level of lactate import. The intracellular acetate pool (about 170 mM) peaked as the fast intracellular lactate conversion ceased. Lactate and acetate were produced as end-products of glucose metabolism, but after glucose depletion lactate was converted into equimolar amounts of acetate. At the end of the experiment, approximately 70% of the glucose was recovered as acetate. This is a remarkable observation, as aerobically *S. aureus* is normally recognized as an acetate consumer [12]; [14].

#### Semi-aerobic glucose metabolism

**Figure 3:**
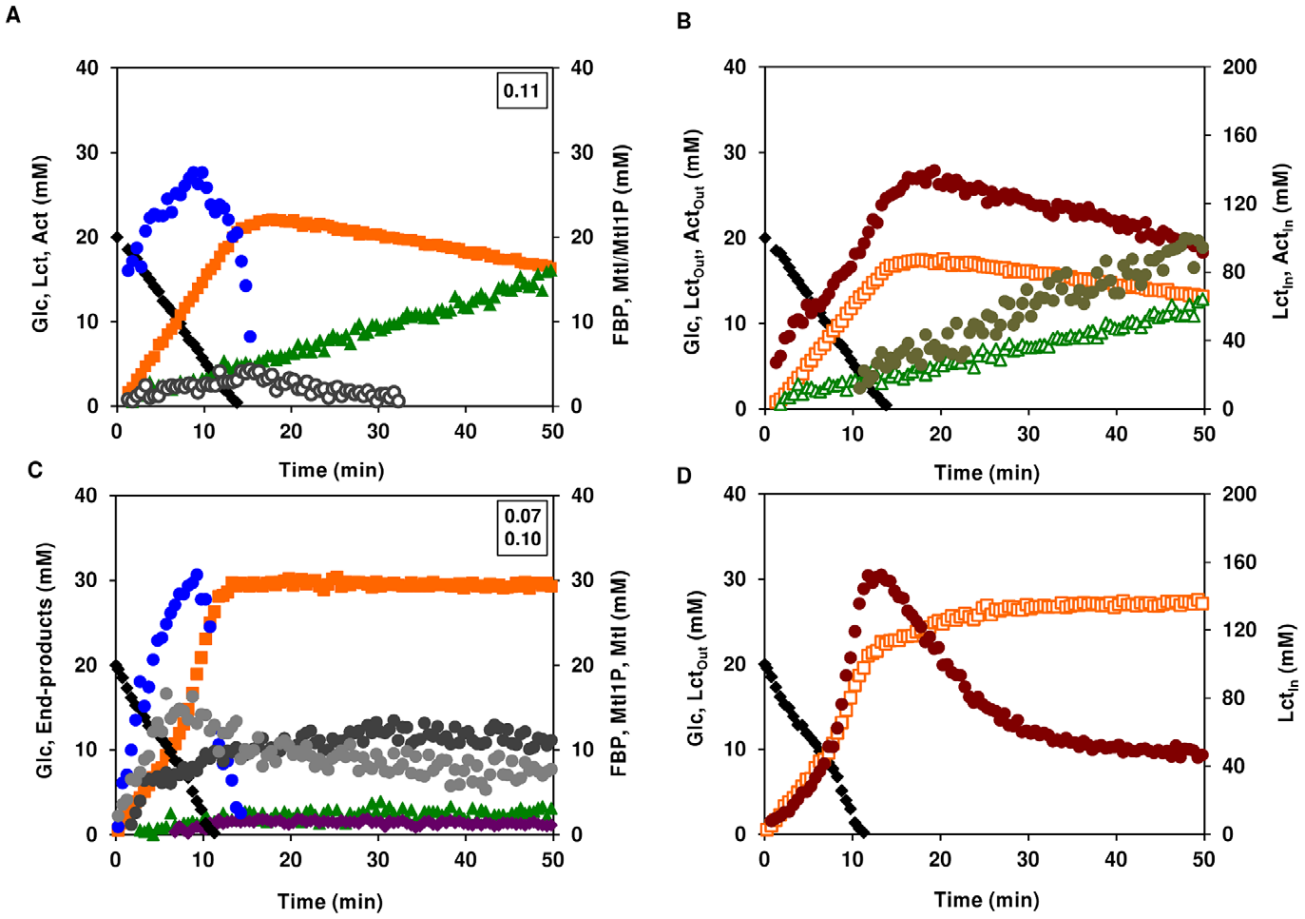
Effect of oxygen limitation on glucose metabolism in aerobically-grown *S. aureus*. Kinetics of [1-^13^C]glucose (20 mM) consumption, end-products formation and phosphorylated intermediates in resting cells of aerobically-grown *S. aureus* COL-S under semi-aerobic (A) and anaerobic (C) conditions. Maximal glucose consumption rates (µmol min^−1^ mg^−1^ of protein) are boxed in the upper-right corners. Dynamics of intra- and extracellular pools of lactate and acetate under semi-aerobic (B) and anaerobic (D) conditions; Glc is also shown for the sake of clarity. Symbols: (black diamond), glucose; (orange square), total lactate; (green triangle), total acetate; (bordeaux diamond), ethanol; (blue circle), FBP; (open orange square) extracellular lactate; (brown circle) intracellular lactate; (open green triangle) extracellular acetate; (olive circle) intracellular acetate; (grey circle) Mtl1P, (dark grey circle) mannitol; (open circle) Mtl1P+mannitol.

The metabolism of glucose was studied by *in vivo* ^13^C-NMR in dense suspensions of aerobically-grown *S. aureus* cells sparged with air (20.95% oxygen) (Fig. 3A and B). Decreasing five times the oxygen concentration, by switching from pure oxygen to air, led to considerably altered glucose metabolism (compare Fig. 2B with 3A). In particular, the rate of glucose consumption was 3-fold lower, and the products of glucose metabolism changed. The carbon recovered as end-products accounted for 86% of the supplied glucose. Both the increase in the yield of end-products (from 70 to 86%) and the switch from acetate to lactate indicate that respiration is limited by oxygen availability. Lactate was the major product from glucose metabolism, but acetate was also formed. At the onset of glucose depletion the total lactate concentration (*circa* 21 mM) was 3.7-fold higher than that of total acetate (approx. 6 mM). With glucose exhaustion, lactate was slowly converted to acetate. In contrast to fully oxygenated cells, the decline of the intracellular lactate pool was slow (25 times lower than in fully oxygenated cells) and steady, but the extracellular lactate was used at a similar rate. These findings suggest that oxygen is a limiting factor for internal lactate metabolism, but not for the transport step. The profiles of intracellular lactate and acetate pools accompanied those of the external metabolites, but at the point of glucose exhaustion the intracellular concentrations were 6 to 7-fold higher. Thus, the concentration gradient favours secretion, implying that lactate must be actively taken up in these cells. The FBP accumulation profile resembled that of fully oxygenated cells, except that the maximum concentration was slightly higher (about 27 mM) and occurred when about 2/3 of glucose had been consumed. Additionally, mannitol-1-phosphate (Mtl1P) and/or mannitol also accumulated transiently, peaking at the onset of glucose depletion and decreasing thereafter. Intracellular accumulation of Mtl1P/mannitol had been previously reported, but only during the anaerobic metabolism of glucose by aerobically-grown cells [19].

#### Anaerobic glucose metabolism

Unsurprisingly, we observed production of Mtl1P and mannitol during the anaerobic metabolism of glucose by suspensions of aerobically-grown *S. aureus* COL-S cells (Fig. 3C). Depletion of oxygen led to biphasic glucose consumption kinetics: a first slower phase (0.07±0.01 µmol min^−1 ^mg^−1^ of protein) was followed by an unexpectedly faster phase (approx. 1.5-fold higher). FBP and Mtl1P pools peaked along with the phase transition and were consumed thereafter. FBP decreased to undetectable levels short after glucose depletion, whereas Mtl1P levelled off at a concentration of about 8 mM. Mannitol accumulated up to about 13 mM (intracellular concentration). Lactate was the major product from glucose metabolism, but acetate, and lesser amounts of ethanol and 2,3-butanediol were also formed. The carbon balance was around 94%, a value typical of fermentative metabolism.

Lactate accumulated both intracellularly and in the external medium (Fig. 3D). At the onset of glucose exhaustion, the intracellular lactate reached a maximal concentration of 159±10 mM, subsequently decreasing to a steady concentration of 47±4 mM. The build-up of the extracellular lactate pool mirrored the disappearance of intracellular lactate. Thus, the intracellular decrease is not due to lactate utilization, but merely to secretion to the surrounding medium. Lactate metabolism was not observed in the absence of oxygen.

The *in vivo* NMR time series obtained during the metabolism of glucose in dense suspensions of *S. aureus* COL-S cells grown aerobically clearly showed that central carbon metabolism is dramatically influenced, at least at the metabolic level, by oxygen availability. Under the conditions studied, lactate, but not acetate, metabolism was stimulated by oxygen.

### Anaerobic Glucose Metabolism of Anaerobically-grown *S. aureus* COL-S Cells is Homolactic

**Figure 4:**
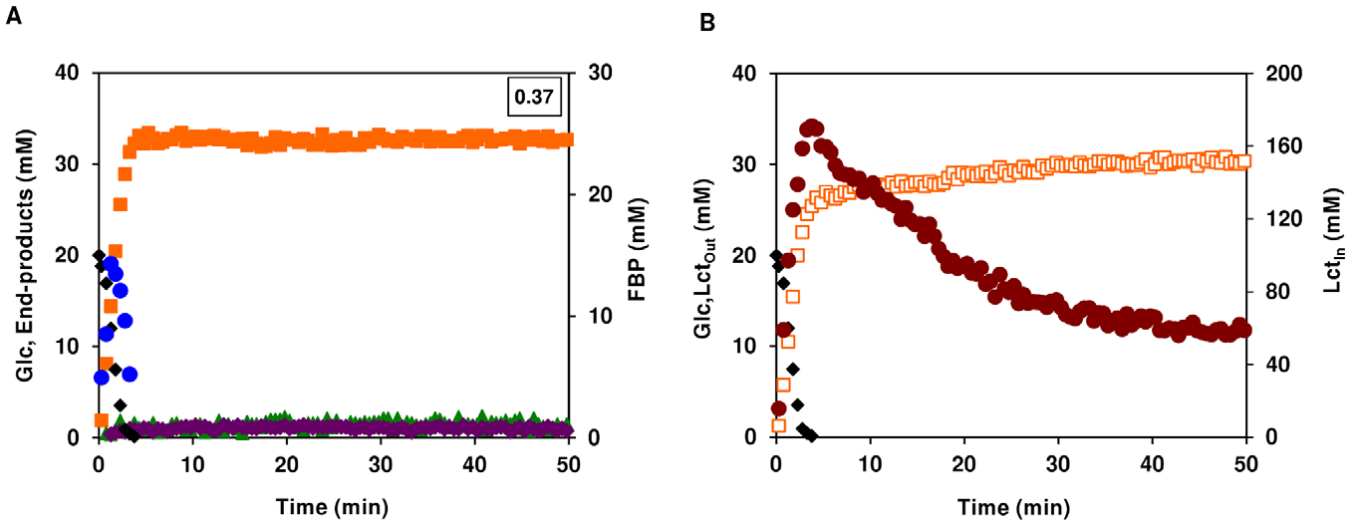
Anaerobic glucose metabolism in anaerobically-grown *S. aureus*. Kinetics of [1-^13^C]glucose (20 mM) consumption, end-products formation and phosphorylated intermediates in resting cells of aerobically-grown *S. aureus* COL-S. Maximal glucose consumption rates (µmol min^−1^ mg^−1^ of protein) are boxed in the upper-right corners (A). Dynamics of intra- and extracellular pools of lactate under anaerobic conditions (B); Glc is also shown for the sake of clarity. Symbols: (black diamond), glucose; (orange square), total lactate; (green triangle), total acetate; (bordeaux diamond), ethanol; (blue circle), FBP; (open orange square) extracellular lactate; (brown circle) intracellular lactate.

A previous study established that an aerobic-anoxic switch led to altered transcript levels of many genes involved in central carbon metabolism [10]. To discriminate between metabolic and transcriptional regulation we ascertained the anaerobic metabolism of glucose by anaerobically-grown *S. aureus* cells (Fig. 4). The glucose consumption was consistently higher in cells grown anaerobically than in cells grown aerobically (0.37±0.03 compared to 0.33±0.03 µmol min^−1 ^mg^−1^ of protein). In agreement, during anaerobic growth *S. aureus* COL-S also used glucose at a higher rate than in the presence of oxygen (Table 1). Lactate was the main end-product, accounting for about 82% of the glucose supplied, but minor amounts of acetate, ethanol and 2,3-butanediol were also formed. FBP accumulated transiently to a maximal concentration of 14±1 mM, decreasing to undetectable levels before glucose depletion, and no Mtl1P or mannitol were detected in anaerobically-grown cells. Under these conditions, the intracellular lactate pool peaked (185±14 mM) at the onset of glucose exhaustion, upon which the intracellular lactate was slowly excreted yielding proportional amounts of extracellular lactate (Fig. 4B). Eventually, both pools reached steady concentrations of 62±3 and 31±1 mM for intracellular and extracellular lactate, respectively. The steady-state concentration ratio of intracellular to extracellular lactate (∼1.9–2.0) is similar to that found during the anaerobic metabolism of glucose by aerobically-grown cells.

Our data showed that oxygen mediates direct regulation at the metabolic level, and is in line with an earlier study [10] demonstrating that oxygen plays a pivotal role in regulating the expression of genes involved in central metabolism, namely glycolysis, fermentation and TCA cycle. Combined, the effects resulted in dramatic changes in energy and redox metabolism.

### Lactate is a Carbon Source for the Growth of *S. aureus*

**Figure 5:**
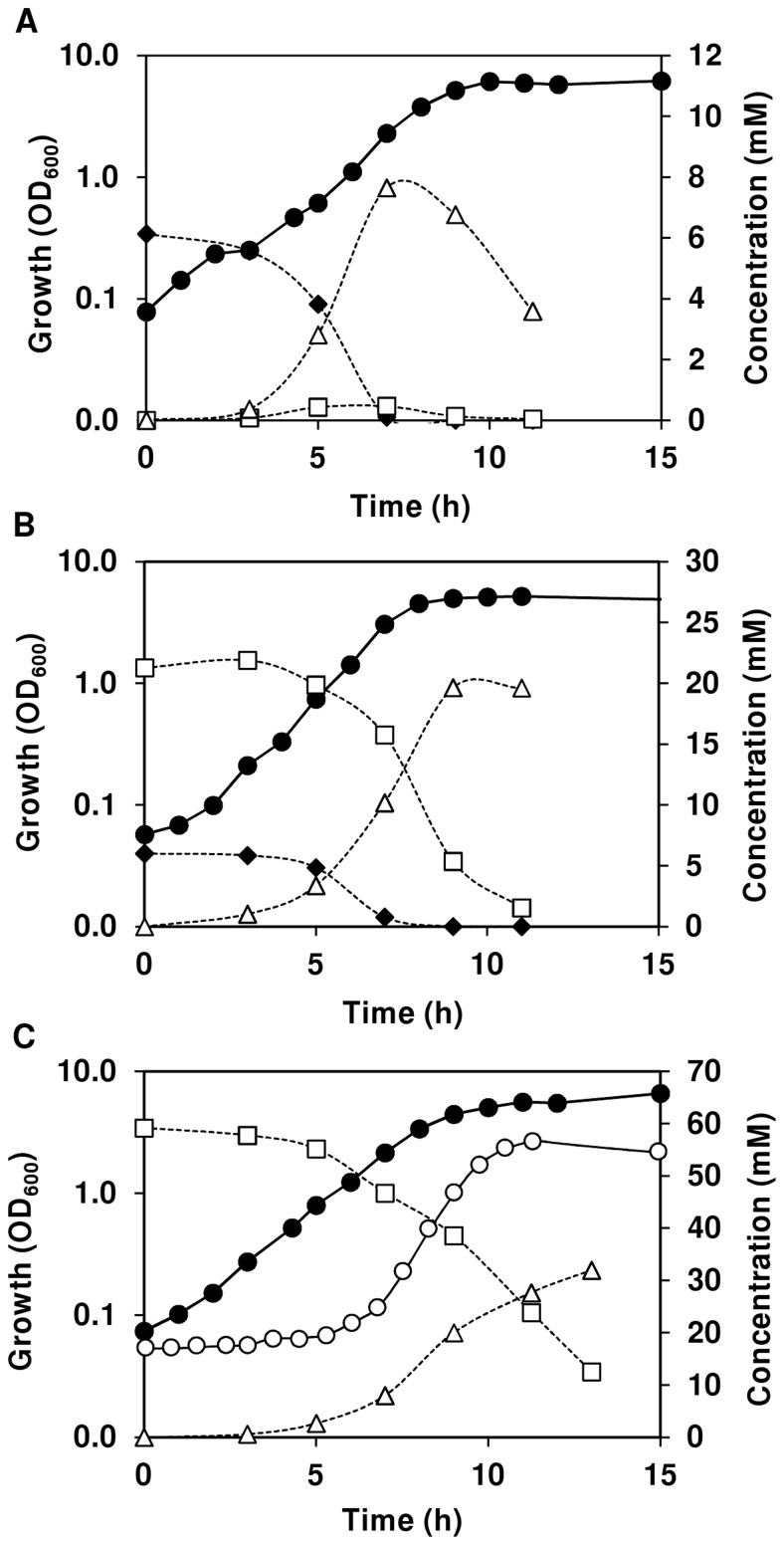
Lactate as a carbon source for aerobic growth of *S. aureus*. Growth, substrate consumption, and end-product formation by *S. aureus* COL-S using a limiting glucose concentration (A), a glucose/lactate mixture (B), and lactate (C) as carbon sources in CDM and under aerobic conditions. Symbols: (black diamond), glucose; (open square), lactate; (open triangle), acetate; (black circle), growth in CDM with lactate (58 mM); (open circle) growth in CDM without additional carbon source. Data from a representative experiment where the error in each point ≤10%.

Intrigued by the *in vivo* ^13^C-NMR results, and in particular by the ability of non-growing *S. aureus* cells to efficiently metabolize lactate in the presence of oxygen, we sought to assess whether this metabolic trait would also occur during growth. In resting cells, lactate was only used after glucose depletion. Thus, we examined the growth pattern of *S. aureus* COL-S under a limiting glucose concentration (6.2 mM) in CDM and aerobic conditions as above (Fig. 5A and Table 1). Decreasing the glucose concentration by about 10-fold was not reflected on the growth curve shape or growth rate, but imposed a modest decrease in the maximal biomass reached (Table 1; Fig. 1A and 5A). In contrast to growth in the presence of excess glucose, the sugar was exhausted after 7 h in exponential phase. While glucose was available, lactate and acetate accumulated in the medium, but were subsequently consumed after depletion of the hexose. Interestingly, lactate consumption and depletion preceded that of acetate. However, under these conditions lactate was a minor product (maximal yield 0.07), hence its contribution for catabolism and growth was close to negligible. To overcome this problem we proceeded to study growth of *S. aureus* in mixtures of glucose and lactate (Fig. 5B). To our surprise, lactate was efficiently used and no clear preference for glucose was observed. In fact, glucose and lactate were metabolized simultaneously forming mainly acetate as end-product (carbon recovery of about 70%). Interestingly, lactate seemed to positively affect the maximal rate of glucose consumption (Table 1). These promising results led us to investigate whether lactate could be used as sole carbon source for growth of S. *aureus* COL-S. As shown in Fig. 5C, under aerobic conditions *S. aureus* can grow on lactate alone at a maximal growth rate of 0.72±0.02 h^−1^. The main end-product of lactate metabolism was acetate and the carbon recovery was about 78%. Under these conditions, *S. aureus* reached a maximal biomass of 6.7±0.2, which is only slightly lower than that when non-limiting glucose was the substrate. This growth pattern was firmly associated with the presence of lactate in the medium, since in CDM without lactate (or glucose) growth was characterized by a long lag phase (about 6 h, as compared to less than 15 min in CDM with lactate)and much lower biomass (OD_600_, 2.6±0.2, Fig. 5C). In the absence of lactate (or glucose) acetate (∼1 mM, at time-point 12 h) was the main product detected by the HPLC method herein used, which detects organic acids and some monosaccharides and alcohols.

Herein, we demonstrated that *S. aureus* COL-S possesses the ability to use lactate as carbon source for growth and that it shows no preference for glucose when lactate and oxygen are also available.

## Discussion

*S. aureus* is a common resident of the human body, but also an opportunistic pathogen responsible for a variety of diseases in humans that range in severity from mild to fatal. However, survival and proliferation of *S. aureus* in different host niches is largely determined by its ability to perceive and rapidly adapt to fluctuating environmental parameters. One such varying factor is oxygen, and in the progression from colonization to endogenous infection, *S. aureus* presumably migrates from fully aerobic to near anoxic milieus. In this work, the response of *S. aureus* to oxygen deprivation was examined as a means to provide insights into the metabolic mechanisms used by the pathogen in internal anoxic host niches.

We took advantage of the non-invasive and analytical power of NMR to generate online metabolic information during the consumption of glucose (Figs. 2–4). In an elegant study from the 1980’s, this technique was used to probe several aspects of glucose metabolism in *S. aureus* [19]. Although relevant qualitative information was obtained (discussed below), most experiments were conducted under suboptimal conditions of temperature (20°C or less) and pH (6.0). Moreover, the reliable quantification of metabolites was possibly hampered by the extensive line broadening of intracellular resonances attributed to the accumulation of manganese, which is present in elevated amounts in the brain heart infusion medium used for growth. To minimize this effect we used a modified CDM devoid of paramagnetic ions that, under aerobic conditions, sustained growth up to OD_600_ of 6.1 for a glucose concentration of 6.2 mM, as compared to ODs of around 2.2–2.5 with 10 mM glucose reported in the literature [16]; [21]; [22].

**Figure 6:**
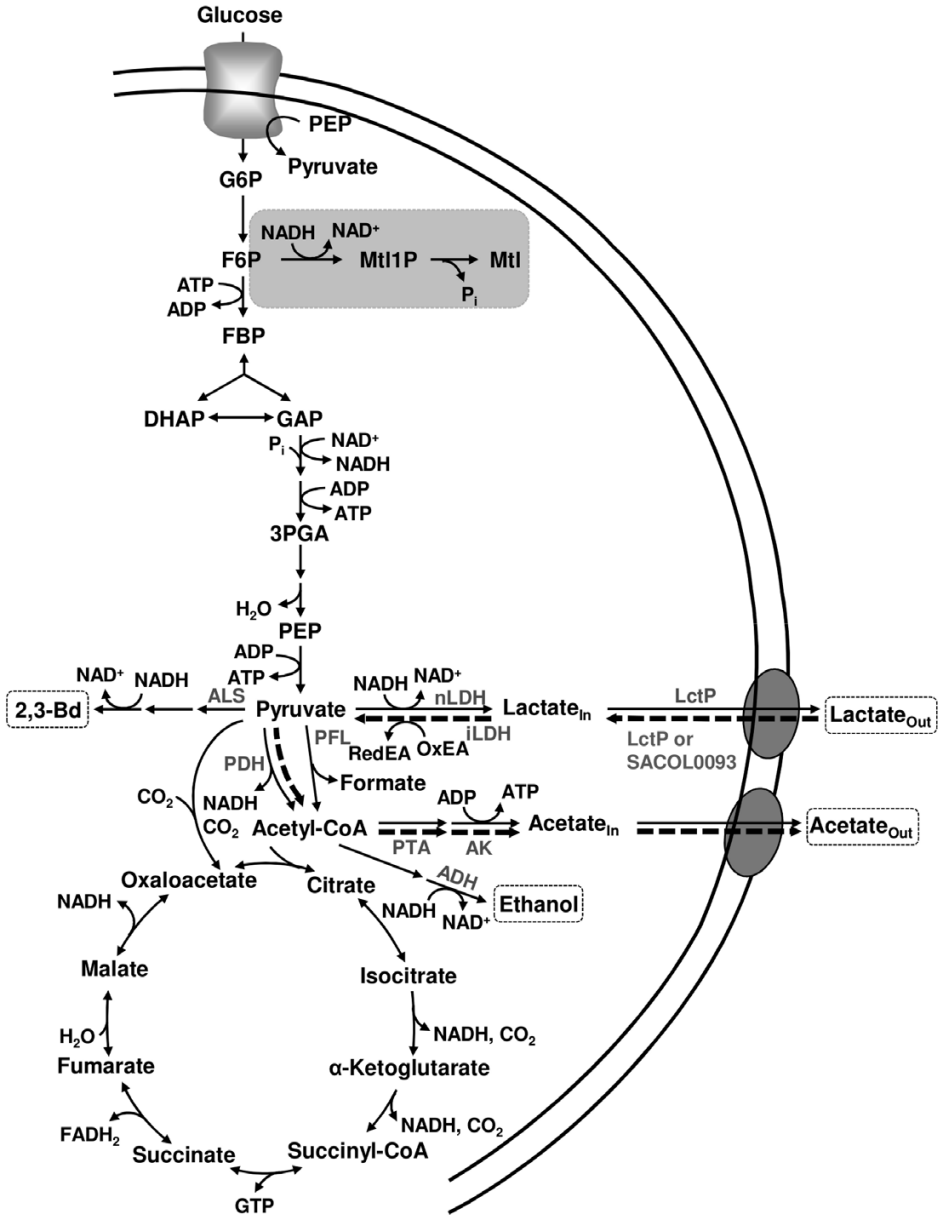
Scheme of central carbon metabolism in *S. aureus* COL-S. Glycolysis, tricarboxylic acid (TCA) cycle, pyruvate metabolism and pathway for mannitol production are depicted. Production of mannitol by aerobically-grown cells under limiting oxygen and anaerobic glucose metabolism is highlighted in a grey box. The newly proposed pathway for the metabolism of lactate under aerobic conditions is indicated by thick dashed arrows. Anaerobic end-products are shown in dashed boxes. Enzymes involved in pyruvate metabolism and aerobic lactate are shown in grey. LctP, lactate permease SACOL2363; nLDH, NAD^+^-dependent lactate dehydrogenase; iLDH, NAD^+^-independent lactate dehydrogenase; PDH, pyruvate dehydrogenase complex; PFL, pyruvate formate-lyase; ALS, α-acetolactate synthase; ADH, alcohol dehydrogenas; PTA, phosphotransacetylase; AK, acetate kinase.

The direct (metabolic) effect of oxygen or its deprivation on glucose metabolism was assessed in cells grown aerobically (Fig. 1A), and with similar cell machinery at the transcript and protein levels. *S. aureus* COL-S cells grown in the presence of oxygen (aerobic growth), slowed down the utilization of glucose with decreasing oxygen concentrations. This behaviour reflects the metabolic shift from aerobic respiration to the less efficient fermentative metabolism without prior transcriptional adaptation. The imposed substrate limitation (O_2_ depletion) renders the aerobic respiratory chain limiting or inoperative, thus for glycolysis to proceed, activity of alternative pathways for NAD^+^ recycling is required. To fulfil the redox balance, *S. aureus* deviated from CO_2_ and acetate to lactate production, as denoted by a increase in the maximal lactate yield from aerobic to anaerobic metabolism (Y_Lct/Glc_: 0.64 aerobic; 1.1, semi-aerobic; 1.5 anaerobic). The dramatic metabolic shift to lactate fermentation was accompanied by formation of mannitol and/or Mtl1P (Fig. 6). Accumulation of mannitol in *S. aureus* was first reported by Edwards and co-workers [23], who speculated that this metabolite could perform an osmoregulatory function, whereas Mtl1P most likely served as an electron sink. In homofermentative lactic acid bacteria, formation of these polyols has been rationalized as an alternative mean to regenerate NAD^+^ [24]; [25]. This interpretation is consistent with the high pressure endured by aerobically-grown *S. aureus* cells to maintain a balanced NAD^+^/NADH ratio when oxygen becomes limiting.

Decreasing oxygen availability causes a metabolic jamming of glycolysis, as indicated by the increasing concentrations of FBP (Fig. 2A, 3A and 3C), and this ultimately translates in lower rates of glucose utilization. The glycolytic key enzyme glyceraldehyde 3-phosphate dehydrogenase is inhibited by NADH [26]–[28], which accumulates under oxygen-deprived conditions. Hence, it is reasonable to argue that the negative effect of oxygen deprivation on the glucose utilization rate can be partially due to inhibition of glyceraldehyde 3-phosphate dehydrogenase by NADH. An intriguing observation was the biphasic glucose consumption displayed by aerobically-grown *S. aureus* COL-S metabolizing glucose anaerobically. Although, a complete explanation to this phenomenon cannot be put forward, accumulation of Mtl1P was halted concomitantly with the switch to faster substrate consumption, which indicates alleviation of the pressure to regenerate NAD^+^. The appearance of ethanol (Fig. 3C), a highly reduced end-product of glucose fermentation, and the substantial increase in the rate of lactate production, as denoted by the strong inflexion in the intracellular pool (Fig. 3D), are in line with this view. However, the molecular signal promoting alcohol and lactate dehydrogenase activities remains elusive. Activation of lactate dehydrogenase by glycolytic metabolites, and in particular FBP, is well known [29]. Nonetheless, the intracellular concentrations of FBP in *S. aureus* (20–30 mM) are greatly beyond the reported *K*_act_ (micromolar range) of FBP for lactate dehydrogenases [30]. In summary, our data shows that aerobically-grown *S. aureus* is equipped with the molecular machinery required to cope with oxygen-deprivation. The metabolic priority is the maintenance of a balanced NAD^+^/NADH ratio, which is achieved by slowing down glucose utilization, and thus NADH production, and resorting to alternative pathways for NAD^+^ regeneration.

When subjected to anaerobic growth, *S. aureus* counterbalances the loss of an operative aerobic respiratory chain by inducing transcription of glycolytic and fermentative genes, while repressing genes involved in the TCA cycle and the pyruvate dehydrogenase complex [10]; [13]; [15]; [31]. In this study, the highest rate of glucose utilization was detected for anaerobically-grown *S. aureus* COL-S cells metabolizing glucose anaerobically, a finding indicative of high glycolytic flux. In agreement, the FBP concentration is halved when compared to aerobically-grown cells, and mannitol/Mtl1P were not detected. These results further support the idea that mannitol production provides a surrogate pathway for NAD^+^ recycling. Lactate was the main fermentation product, but ethanol, 2,3-butanediol and alanine were also detected, and their concentrations were doubled when compared to those resulting from anaerobic glucose metabolism by aerobically-grown *S. aureus*. Our metabolic data are in line with the altered gene and protein levels previously reported [10]; [13]; [15]; [31]. The identification in *S. aureus* of Rex, a Rex-family repressor, which senses the redox status of the cell, was a major breakthrough to the understanding of the mechanisms underlying the genetic switch from aerobic to anaerobic growth [13]. The binding of Rex to specific DNA motifs, and thus its repressing activity, is enhanced by NAD^+^ and diminished by NADH. The NAD^+^/NADH ratio of *S. aureus* is approximately 2-fold higher during aerobic growth as compared to anaerobic growth [15]. The lower anaerobic NAD^+^/NADH ratio probably causes inhibition/inactivation of Rex activity, and thus de-repression of Rex-regulated genes. The Rex regulon encompasses genes encoding the main lactate dehydrogenase (*ldh1*), a lactate permease (*lctP*), alanine dehydrogenase, and genes involved in formate and ethanol formation as well as nitrate/nitrite respiration (anaerobic respiration). Hence, in *S. aureus* the NAD^+^/NADH ratio is a main signal conduit and Rex is the effector in the regulation of anaerobic metabolic pathways, albeit the existence of other auxiliary regulators cannot be discarded [13]. In summary, adaption to fluctuating oxygen environments seems to be of chief importance to *S. aureus.* Also curious to note was that both the metabolic and genetic strategies to withstand oxygen-deprivation seem to overlap and thus add to each other.

The *in vivo* NMR technique used in this study was especially valuable to provide unique information on the intracellular and extracellular pools of lactate and acetate. The ability of *S. aureus* to metabolize acetate aerobically has been recognized for over half a century [14]. One of the most striking findings in our study is that lactate is also efficiently utilized by *S. aureus* (Fig. 2). In fact, lactate is converted to equimolar amounts of acetate, which in our setting is not further used by *S. aureus* COL-S (Fig. 2 and 6). To dissect the biochemistry of this process we took advantage of the appearance of disparate resonances corresponding to intra- and extracellular pools of lactate and acetate in ^13^C-NMR spectra of living cells of *S. aureus.* This enabled us to determine the distribution of the weak organic acids in the internal and external spaces. We found a concentration gradient that favours the intracellular compartment (intracellular concentration is always higher than the external concentration), *i.e.*, the rate of intracellular lactate accumulation (1.5 to 3.5 µmol min^−1^ mg^−1^ of protein, depending on the experimental condition) was swifter than the efflux (0.2–0.4 µmol min^−1^ mg^−1^ of protein) as calculated from the decaying slope of intracellular lactate curves during anaerobic metabolism. Moreover, the internal/external ratio was four fold higher in the presence of oxygen, suggesting a tighter coupling between production and efflux under anaerobic conditions. This coupling could be accomplished through Rex-mediated adjustment of lactate dehydrogenase and permease gene expression [13]; [19]. In an earlier work, oxygen was shown to influence the internal/external distribution of lactate, *i.e.*, during an oxygenation cycle influx of lactate occurred and was followed by a net efflux during anaerobic incubation [19], but stimulation of lactate catabolism by oxygen as observed by us was not reported. Our data also shows that lactate utilization is impaired by oxygen limitation (compare Fig. 2C with 3B), and totally abolished under anaerobic conditions, consistent with inactivation of the TCA cycle. Comparison of the dynamics of intracellular and extracellular lactate under aerobic and semi-aerobic conditions (Fig. 2C and 3B) allowed us to conclude that rather than the import step (average influx rate of 0.01–0.02 µmol min^−1^ mg^−1^ of protein, the drop in oxygen concentration affects the intracellular conversion of lactate (oxidation to pyruvate or subsequent conversions to acetate). Indeed, the maximal rate of intracellular lactate utilization is 25-fold higher under aerobiosis than under semi-aerobic conditions (2.5 µmol min^−1^ mg^−1^ of protein compared to 0.1 µmol min^−1^ mg^−1^ of protein).

As for the pathway involved in lactate utilization (Fig. 6), we propose an energy-dependent import step to offset the concentration gradient that favours the intracellular compartment (higher intracellular concentrations). Good candidates to catalyze this step are the two LctP type lactate permeases (SACOL0093 and SACOL2363) found in the genome of *S. aureus* COL by homology to the LctP permease (LldP) of *Escherichia coli* [32]. Even though SACOL2363 has been implicated in the secretion of lactate in *S. aureus* [13], a role in lactate import cannot be ruled out as these transporters can function bidirectionally [32]; [33]. The subsequent oxidation of lactate to pyruvate could be catalyzed by NAD^+^-dependent or NAD^+^-independent lactate dehydrogenases. A genome survey of *S. aureus* COL revealed two NAD^+^-dependent L-lactate dehydrogenases (*ldh1* [13] and the NO-inducible *ldh2* [15]), and two D-lactate dehydrogenases (SACOL2535 and SACOL2574). Although NAD^+^-dependent dehydrogenases are known to mediate the aerobic lactate oxidation in the fermentative bacterium *Lactobacillus plantarum* [34], this is a rare event in nature. Indeed, in respiratory organisms including *E. coli* and *Corynebacterium glutamicum* the aerobic conversion of lactate to pyruvate is catalyzed, in a flavin-dependent mechanism, by an NAD^+^-independent dehydrogenase that uses a membrane quinone as external acceptor [33]; [35]. Homologs of the *C. glutamicum* L-lactate:quinone oxidoreductase were not found in the genome sequence of *S. aureus*, which possesses, however, two 2-hydroxy acid-quinone:oxidoreductase genes (SACOL2362 and SACOL2623 annotated as malate:quinone oxidoreductases) that could undertake this function as proposed by Tynecka *et al.* [36]. Experimental verification of their role in lactate oxidation is out of the scope of this work. Ensuing conversion of pyruvate to acetate occurs most likely through the action of the pyruvate dehydrogenase complex, phosphotransacetylase and acetate kinase (Fig. 6).

Fostered by the efficient aerobic lactate metabolism displayed by resting cells of *S. aureus,* we set out to examine whether growing cells could also use lactate. We found that when glucose becomes limiting, *S. aureus* COL-S maintains exponential growth by switching to the aerobic utilization of lactate, rather than utilizing the overflow acetate. Oxidation of lactate to acetate provides a cost-effective means of generating ATP at the level of acetate kinase (Fig. 6). Reduction of the external lactate concentrations in *S. aureus* cultures in post-exponential phase or by succinate dehydrogenase mutants has been described before [16]; [37], but we are unaware of reports suggesting usage of lactate to sustain growth. Additionally, we showed that in mixtures of glucose and lactate both metabolites were used without a clear preference for any of them. Finally, to our knowledge, we are the first to demonstrate the ability of *S. aureus* to grow on lactate in the absence of an added sugar source. We propose that this newly discovered feature, the aerobic oxidation of lactate, might present an ecological advantage for *S. aureus* in host niches such as the skin and the nasal cavity where the oxygen concentrations are high. In these ecological niches, the concentration of free monosaccharides, the preferred carbohydrates of most bacteria, is low. *S. aureus* cohabits with several fermentative commensal and pathogenic bacteria, such as streptococci, whose major end-product is lactate. Thus, the availability to use a waste product of bacterial metabolism endows *S. aureus* with a metabolic flexibility missing in fermentative bacteria inhabiting the same niches, and thus competing for nutrients. In this light, the ability to use lactate for metabolism and growth might confer a competitive advantage and improved fitness of *S. aureus* in the host. Importantly, this view is corroborated by a study showing that lactate oxidation promoted successful colonization and survival of the human pathogen *Neisseria gonorrhoeae* in the genital tracts of mice [38].

## Materials and Methods

### Bacterial Strains and Growth Conditions

In this study *Staphylococcus aureus* COL-S [39], a methicillin resistant strain lacking the SCC cassette that includes the *mecA* gene, was used. Routinely, *S. aureus* was cultivated in Tryptic Soy Broth medium (Difco Laboratories), and bacterial stocks kept at −80°C in 30% (vol/vol) glycerol. For *in vivo* NMR studies of metabolism, we developed a chemically defined medium (CDM) considering the subsequent criteria: (i) nutrient requirements of *S. aureus*, (ii) composition of available CDMs for *Bacilli*, and (iii) need to limit the concentration of paramagnetic ions (for *in vivo* NMR) while sustaining high biomass. The composition of the CDM for *S. aureus* is as follows: 3.4 g l^−1^ KH_2_PO_4_, 2.9 g l^−1^ K_2_HPO_4_, 0.7 g l^−1^ di-ammonium citrate, 1.3 g l^−1^ sodium acetate, 0.26 g l^−1^ NaHCO_3_, 0.7 g l^−1^ MgSO_4_. 7H_2_O, 7 mg l^−1^ CaCl_2_. 2H_2_O, 100 mg l^−1^ of the amino acids L-alanine, L-arginine, aspartic acid, potassium L-glutamate, L-glutamine, glycine, L-histidine hydrochloride, L-isoleucine, L-leucine, L-lysine, L-methionine, phenylalanine, L-proline, L-serine, L-tryptophan, L-valine and trans-4-hydroxy-L-proline, 350 mg l^−1^ L-tyrosine, 1.1 g l^−1^ L-cysteine hydrochloride, 200 mg l^−1^ of threonine, 20 mg l^−1^ of the nitrogenous bases adenine, guanine and uracil, 2 mg l^−1^ 4-*p*-aminobenzoic acid, 2 mg l^−1^ D-biotin, 8 mg l^−1^ folic acid, 10 mg l^−1^ nicotinamide, 20 mg l^−1^ calcium D-pantothenate, 10 mg l^−1^ pyridoxine hydrochloride, 10 mg l^−1^ pyridoxal hydrochloride, 20 mg l^−1^ riboflavin, 10 mg l^−1^ thiamine hydrochloride and 1 mg l^−1^ vitamin B12. The medium was prepared in bi-distilled water (Millipore E-POD), and the pH adjusted to 7.0.

Cultivations were performed at 37°C, without pH control (initial pH 7.0), and glucose (65 mM) used as carbon source, unless stated otherwise. For aerobic conditions cells were grown in shake flasks (CDM volume 1/5 of the flasks total capacity) in an orbital shaker (AGITORB 200, Aralab) at 200 rpm, while nearly anaerobic conditions were maintained by growing the cells in static rubber-stoppered bottles (100-ml). To assess whether a medium volume of 1/5 of the flask total capacity ensured aerobic growth, growth in baffled shake flasks (1/5 volume to flask ratio) or 1/8 volume to flask ratios was also tested. Cultures were initiated by addition of a pre-culture, which was grown overnight for 14 h under aerobic conditions in glucose-CDM, to an initial optical density at 600 nm (OD_600_) of about 0.06–0.07. Growth profiles in CDM supplemented with 1/10 of the standard glucose concentration (1/10 glucose, 6.3±0.2 mM), 1/10 glucose plus lactate (23.0±2.4 mM), or lactate (58.7±2.4 mM) were also obtained. Growth was monitored by measuring the OD_600_. Specific growth rates (μ) were calculated through linear regressions of the plots of ln(OD_600_) versus time during the exponential growth phase.

### Quantification of Fermentation Products during Growth

Samples (2 ml) were collected at different time-points during growth, centrifuged (16100×*g*, 5 min, 4°C), filtered (0.22 µm), and the supernatant solutions were stored at −20°C until analysed by high performance liquid chromatography (HPLC). Fermentation substrates and products were quantified by HPLC using a HPX-87H anion-exchange column (Bio-Rad Laboratories, Inc.) and a refractive index detector (Shodex RI-101, Showa Denko K.K.) at 60°C with 5 mM H_2_SO_4_ as the elution fluid and a flow rate of 0.5 ml min^−1^. The Chromeleon® software was used for data treatment as described before [40]. The values reported are averages of at least two independent experiments. Under anaerobic conditions the ATP yield was calculated as the ratio of ATP produced to glucose consumed. The global yields of ATP were calculated from the fermentation products determined at the time-point of growth arrest assuming that all ATP was synthesized by substrate-level phosphorylation. A factor of 0.15, determined from a dry weight (DW, mg ml^−1^) versus OD_600_ curve, was used to convert OD_600_ into dry weight (mg biomass ml^−1^).

### *In vivo* ^13^C-NMR Experiments

Cells grown in CDM medium supplemented with glucose (1.1% wt/vol) and harvested in the late-logarithmic phase of growth (OD_600_ of 2.2 or 0.8 for aerobic and anaerobic growth, respectively) were centrifuged, washed twice, and suspended to a concentration of approximately OD_600_ of 200 (DW of about 30 mg ml^−1^) in 100 mM KP_i_ (pH 7.0) with 6% (vol/vol) D_2_O. NMR experiments were performed using a 10-mm NMR tube containing 3 ml of cell suspension. To avoid settling down of the cells and ensure an adequate supply of gases to the cell suspension an air-lift system was used inside the NMR tube [41]. To make the system anaerobic, argon was bubbled through the air-lift system 10 min before and continuously after acquisition was started. For aerobic experiments a similar set-up was used, but air (semi-aerobic or limited oxygen) or pure oxygen (aerobic or fully oxygenated) was used. Glucose (20 mM) specifically labelled in carbon 1 was added at time 0 min and spectra acquired sequentially after its addition. At the end of the *in vivo* NMR experiment the cell suspension was passed through a French press: the resulting cell extract was incubated at 80°C (10 min) in a stoppered tube, cooled down on ice and cell debris and denatured macromolecules were removed by centrifugation. The supernatant (NMR-extract) was used for quantification of end-products and minor metabolites as described below. Although individual experiments are illustrated in each figure, each type of *in vivo* NMR experiment was repeated at least twice and the results were highly reproducible. The values reported are averages of two to four experiments and the accuracy varied from 2% (end-products) to 15% in the case of intracellular metabolites with concentrations below 5 mM.

### Quantification of Products by NMR

Lactate and acetate were quantified in NMR-extracts by ^1^H-NMR [42]. Formic acid (sodium salt) was added to the samples and used as an internal concentration standard. The concentration of minor products (ethanol, mannitol, alanine and 2,3-butanediol) and metabolic intermediates that remained inside the cells (Mtl1P) was determined from the analysis of ^13^C spectra of NMR-extracts as described by Neves *et al.* [42]. The concentration of labelled lactate (or acetate when lactate was depleted) determined by ^1^H-NMR was used as a standard to calculate the concentration of the other metabolites in the sample.

### Quantification of Intracellular Metabolites in Living Cells by ^13^C-NMR

Due to the fast pulsing conditions used for acquiring *in vivo* ^13^C-spectra, correction factors have to be determined to convert peak intensities into concentrations. The correction factor for the resonances due to C_1_ and C_6_ of FBP (0.30±0.02), Mtl1P (0.35±0.01) lactate (0.60±0.01) and acetate (1.81±0.03) were determined from the acquisition of fully and partially relaxed spectra of an NMR-extract to which the metabolites were added to a final concentration of 30 mM. The quantitative kinetic data for intracellular metabolites were calculated from the areas of the relevant resonances, by applying the correction factors and comparing with the intensity of the lactate resonance (or acetate when lactate was depleted) in the last spectrum of the sequence. Partial overlapping of the resonances due to intracellular and extracellular lactate and acetate hampered direct integration. Therefore, to estimate the concentrations of intracellular and extracellular lactate and acetate, deconvolution of the relevant spectral region was performed by fitting the sum of two Lorentzian functions using Matlab V7.1 (The Math Works, Natick, MA, USA). For calculation of the correction factors, ^13^C-NMR spectra were acquired with a 60° flip angle and a recycle delay of 1.5 s (saturating conditions) or 60.5 s (relaxed conditions). Average values derived from two independent experiments were used. The lower limit for *in vivo* NMR detection of intracellular metabolites was 3–4 mM. Intracellular metabolite concentrations were calculated using a value of 1.55 µl mg^−1^ of dry weight for the intracellular volume of *S. aureus* [19].

### NMR Spectroscopy

Carbon-13 spectra were acquired at 125.77 MHz on a Bruker AVANCE II 500 MHz spectrometer (Bruker BioSpin GmbH). All *in vivo* experiments were run using a quadruple nuclei probe head at 37°C as described elsewhere [42]. The acquisition parameters were as follows: spectral width, 30 kHz; pulse width, 9 µs (60° flip angle); data size, 32K; recycle delay, 1.5 s; number of transients, 20. Carbon chemical shifts are referenced to the resonance of external methanol designated at 49.3 ppm.

### Chemicals

[1-^13^C]Glucose (99% enrichment) was obtained from Campro Scientific. Formic acid (sodium salt) was purchased from Merck Sharp & Dohme. All other chemicals were reagent grade.
